# C-Terminal Domain of Aquaporin-5 Is Required to Pass Its Protein Quality Control and Ensure Its Trafficking to Plasma Membrane

**DOI:** 10.3390/ijms222413461

**Published:** 2021-12-15

**Authors:** Shin-ichi Muroi, Yoichiro Isohama

**Affiliations:** Laboratory of Applied Pharmacology, Faculty of Pharmaceutical Sciences, Tokyo University of Science, 2641 Yamazaki, Noda 278-8510, Japan; shin36000903@gmail.com

**Keywords:** aquaporin-5, C-terminal domain, subcellular localization, quality control, autophagy

## Abstract

Aquaporin-5 (AQP5) is selectively expressed in the apical membrane of exocrine glands, such as salivary, lacrimal, and submucosal glands. It is important for the secretory function of exocrine glands because mice with the knockout of AQP5 exhibit a significant reduction in secretion from these glands. Previous reports indicated that the AQP5 C-terminal domain is crucial for the localization of AQP5 at the plasma membrane, but it remains unclear which motif or amino acid residues in the C-terminal domain are essential for this. In this study, we examined the effects of various AQP5 C-terminal deletions or mutations on the expression of AQP5 on the cell surface. AQP5 C-terminal domain mutants did not localize on the plasma membrane, and Leu^262^ was shown to be crucial for AQP5′s plasma membrane localization. The mutants localized in the autophagosome or lysosome and showed decreased protein stability via lysosomal degradation. Taking these findings together, our study suggests that the C-terminal domain is required for AQP5 to pass protein quality control and be trafficked to the plasma membrane.

## 1. Introduction

Aquaporins (AQPs) are water-selective channel proteins that allow the rapid movement of water across the plasma membrane in secretory and adsorptive cells [1]. Among the 13 AQP isoforms, AQP5 is selectively expressed in the apical membrane of exocrine glands [2]. AQP5 knockout (KO) mice show significant reduction of water secretion from the salivary [3] and submucosal glands [4], indicating the importance of AQP5 for exocrine function. In addition, AQP5 dysregulation has been implicated in the states of several diseases, including bronchitis, cystic fibrosis [5], and Sjögren’s syndrome [6,7,8,9]. Because the water pores of AQPs do not have a gating function, the amount of cell surface expression of AQP5 is important for its water transport activity. For example, lipopolysaccharides were shown to increase AQP5 translocalization to the plasma membrane, which is mediated by a p38 MAP kinase-dependent mechanism [10]. Additionally, several reports have shown that an increase in intracellular Ca^2+^ following activation of the M3 acetylcholine receptor stimulates translocation of AQP5 from the intracellular space to the plasma membrane of salivary gland cells [11]. In contrast, nitric oxide was shown to decrease the plasma membrane localization of AQP5 via lipid-raft-dependent endocytosis [12].

Homeostasis of membrane protein localization is maintained by the interaction between the cytosolic domains of membrane proteins and cytosolic proteins such as cytoskeleton components and small GTPase [13,14,15,16,17,18]. AQP5 has four cytosolic domains: N-terminal domain, intracellular loops B and D, and C-terminal domain. AQP5 translocalization to the plasma membrane has been shown to be affected by cAMP and a protein kinase A-dependent mechanism, in which the importance of phosphorylation at Ser^156^ in loop D has been suggested [19]. In addition, the crucial role of AQP5 C-terminal domain for plasma membrane localization has been shown in previous reports using chimera AQP, AQP5/AQP1, and AQP5/AQP8 [20,21]. In the present study, we have investigated the intracellular localization of various AQP5 C-terminal domain mutants.

## 2. Results

### 2.1. C-Terminal Domain Is Required for Plasma Membrane Localization of AQP5

**Figure 1:**
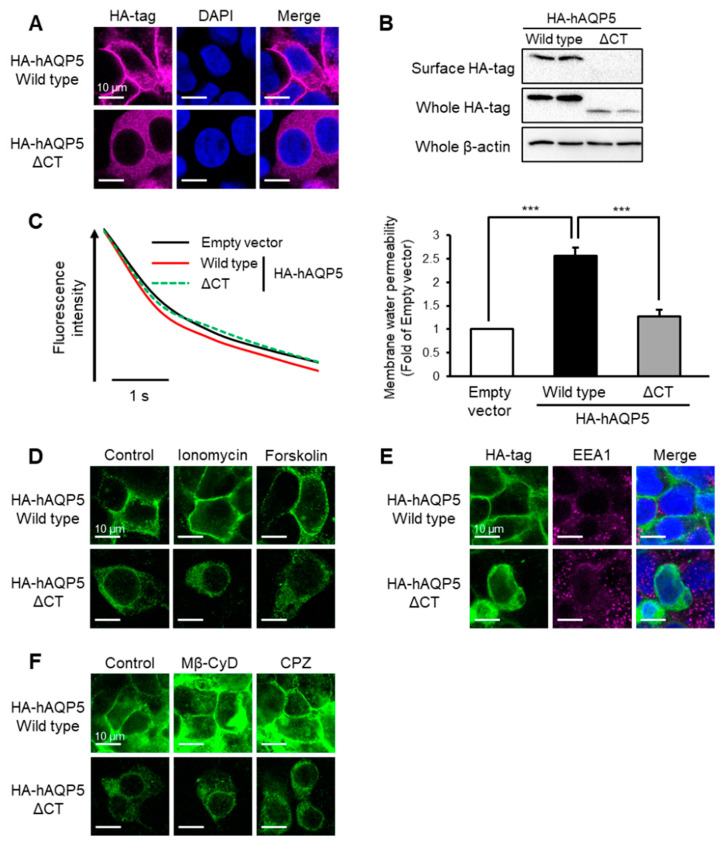
C-terminal domain is required for plasma membrane localization of AQP5. HEK-293 cells transfected with hAQP5-ΔCT were analyzed for the cellular localization of the mutants by immunofluorescence (**A**) and assessed for the level of cell surface mutants by cell surface biotinylation and Western blotting (**B**). Membrane water permeability of CHO-K1 cells transfected with hAQP5-ΔCT was measured using the calcein quenching method at 37 °C. Each data point represents mean ± SE (*n* = 3), *** *p* < 0.001 (**C**). HEK-293 cells transfected with hAQP5-ΔCT were treated with ionomycin (1 µM) or forskolin (10 µM) for 15 min. The cellular localization of the mutants was analyzed by immunofluorescence (**D**). HEK-293 cells transfected with hAQP5-ΔCT were analyzed for the cellular localization of the mutants and EEA1 by immunofluorescence (**E**). HEK-293 cells transfected with hAQP5-ΔCT were treated with methyl-β-cyclodextrin (Mβ-CyD, 1 mM) or chlorpromazine (CPZ, 10 µg/mL) for 12 h. The cellular localization of the mutants was analyzed by immunofluorescence (**F**). Typical data in triplicated experiments are shown.

AQP5 consists of cytoplasmic N- and C-terminal domains. The N-terminal domain (Met^1^ to Lys^12^) is required for protein folding in the endoplasmic reticulum (ER) (data not shown). To determine whether the C-terminal domain of AQP5 is required for its normal localization, we first examined the cellular localization of the hAQP5 mutant that lacks the C-terminal domain (ΔCT, Leu^225^ to Arg^265^) in HEK-293 cells. Intracellular localization was evaluated with immunofluorescence for the hemagglutinin (HA) epitope tag (YPYDVPDYA) linked with the N-terminal of hAQP5. The localization of hAQP5-ΔCT was scattered throughout the cytoplasm, which was different from that of hAQP5-WT (Figure 1A). To determine plasma membrane localization, we then evaluated the amount of cell surface expression using the surface biotinylation method. In the cell surface fraction, hAQP5-ΔCT was not detected, although hAQP5-WT was (Figure 1B). To determine whether this change in AQP5 localization affected cellular function, we measured the membrane water permeability of hAQP5-WT- or -ΔCT-transfected cells. In control cells, fluorescence intensity rapidly decreased in response to hypertonic stimulation. In hAQP5-WT-transfected cells, the decrease in fluorescence intensity occurred more rapidly than that in control cells, but this was not the case in hAQP5-ΔCT-transfected cells (Figure 1C). Then, we also investigated the profile of the abnormal localization of hAQP5-ΔCT. Neither ionomycin nor forskolin treatment, which promote the translocalization of AQP5 to the plasma membrane, changed the cellular localization of hAQP5-ΔCT (Figure 1D). Additionally, we investigated the involvement of endocytosis in the abnormal localization of hAQP5-ΔCT. hAQP5-ΔCT did not colocalize with early endosome antigen-1 (EEA1) (Figure 1E), a marker of early endocytosis, in the cytosol and did not respond to the treatment of methyl-β-cyclodextrin (Mβ-CyD) or chlorpromazine (Figure 1F), both of which are endocytosis inhibitors. These results suggested that the C-terminal domain of AQP5 is required for its plasma membrane localization and that the abnormal distribution of hAQP5-ΔCT is not due to internalization via endocytosis.

### 2.2. Ten Amino Acid Residues between Arg^256^ and Arg^265^ Are Required for Plasma Membrane Localization of AQP5

**Figure 2:**
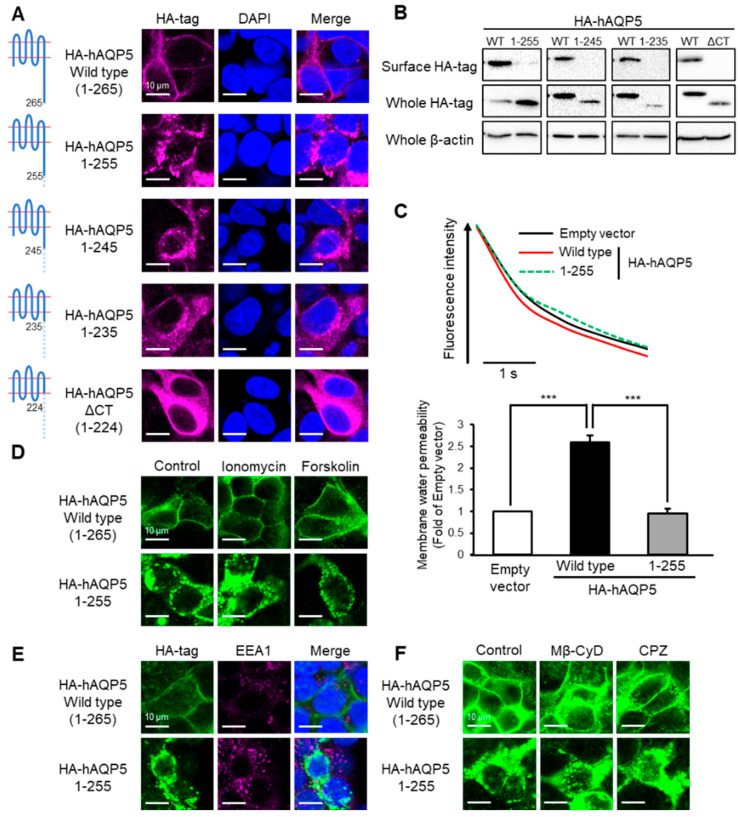
Ten amino acid residues between Arg^256^ and Arg^265^ are required for plasma membrane localization of AQP5. HEK-293 cells transfected with hAQP5 C-terminal deletion mutants were analyzed for the cellular localization of the mutants by immunofluorescence (**A**) and assessed for the level of cell surface mutants by cell surface biotinylation and Western blotting (**B**). Membrane water permeability of CHO-K1 cells transfected with hAQP5 1–255 was measured using the calcein quenching method at 37 °C. Each data point represents mean ± SE (*n* = 3), *** *p* < 0.001 (**C**). HEK-293 cells transfected with hAQP5 1–255 were treated with ionomycin (1 µM) or forskolin (10 µM) for 15 min. The cellular localization of the mutants was analyzed by immunofluorescence (**D**). HEK-293 cells transfected with hAQP5 1–255 were analyzed for the cellular localization of the mutants and EEA1 by immunofluorescence (**E**). HEK-293 cells transfected with hAQP5 1–255 were treated with methyl-β-cyclodextrin (Mβ-CyD, 1 mM) or chlorpromazine (CPZ, 10 µg/mL) for 12 h. The cellular localization of the mutants was analyzed by immunofluorescence (**F**). Typical data in triplicated experiments are shown.

To examine the amino acid residues in the C-terminal domain required for the plasma membrane localization of AQP5 in more detail, we prepared plasmids expressing mutants with deletions of the C-terminal domain, in which increasing numbers of segments of 10 amino acid residues were deleted from the C-terminal end (Table 1). The subcellular localization of the hAQP5 deletion mutants was evaluated with immunofluorescence. The hAQP5 mutant with the deletion of only 10 amino acid residues from the C-terminal end (hAQP5 1–255) localized in cytosol, matching the findings for hAQP5-ΔCT (Figure 2A). Although hAQP5-ΔCT localized throughout the cytoplasm, hAQP5 1–255 localized in the cytoplasm with a punctate pattern. Deleting more amino acid residues did not change the intracellular localization of hAQP5 CT deletion mutants. The amount of cell surface expression of hAQP5 deletion mutants was significantly lower than that of hAQP5-WT (Figure 2B). The levels of membrane water permeability of hAQP5 1–255-transfected cells and control cells were the same, whereas that of hAQP5-WT-transfected cells was higher than that of control cells (Figure 2C). Consistent with the findings for hAQP5-ΔCT, treatment with ionomycin or forskolin did not affect the localization of hAQP5 1–255 (Figure 2D), and endocytosis was shown not to be involved in the abnormal localization of hAQP5 (Figure 2E,F). These results suggested that 10 amino acid residues in the C-terminal domain of AQP5 are required for its localization in the plasma membrane.

### 2.3. Leu^262^ Is Required for Plasma Membrane Localization of AQP5

**Figure 3:**
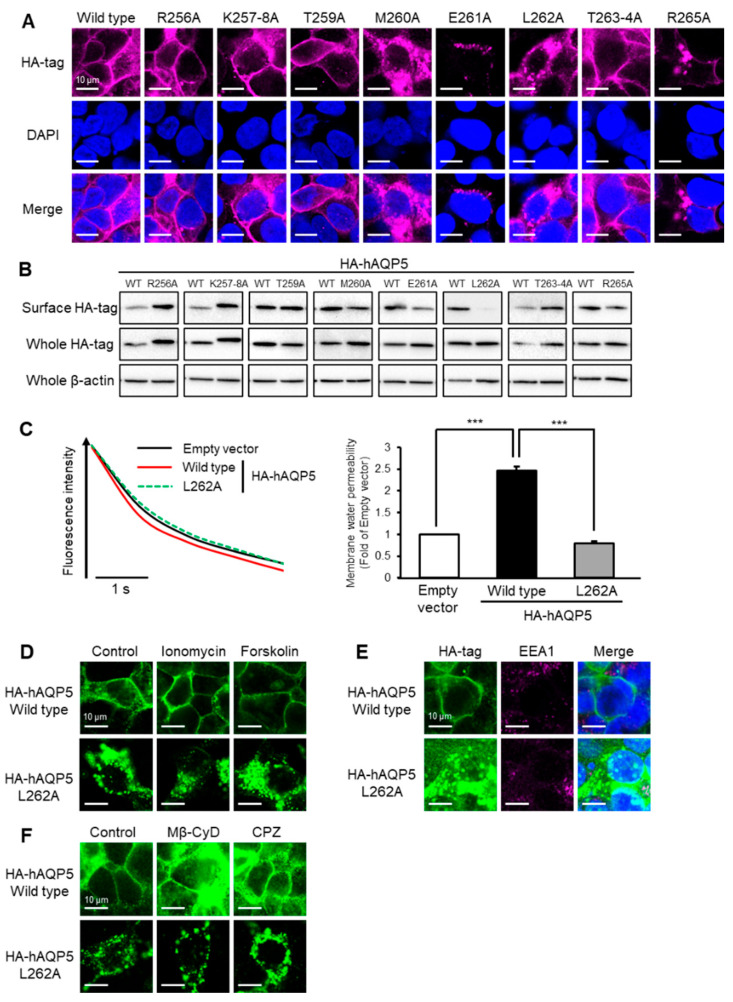
Leu^262^ is required for plasma membrane localization of AQP5. HEK-293 cells transfected with hAQP5 C-terminal point mutants were analyzed for the cellular localization of the mutants by immunofluorescence (**A**) and assessed for the level of cell surface mutants by cell surface biotinylation and Western blotting (**B**). Membrane water permeability of CHO-K1 cells transfected with hAQP5 L262A was measured using the calcein quenching method at 37 °C. Each data point represents mean ± SE (*n* = 3), *** *p* < 0.001 (**C**). HEK-293 cells transfected with hAQP5 L262A were treated with ionomycin (1 µM) or forskolin (10 µM) for 15 min. The cellular localization of the mutants was analyzed by immunofluorescence (**D**). HEK-293 cells transfected with hAQP5 L262A were analyzed for the cellular localization of the mutants and EEA1 by immunofluorescence (**E**). HEK-293 cells transfected with hAQP5 L262A were treated with methyl-β-cyclodextrin (Mβ-CyD, 1 mM) or chlorpromazine (CPZ, 10 µg/mL) for 12 h. The cellular localization of the mutants was analyzed by immunofluorescence (**F**). Typical data in triplicated experiments are shown.

Next, we evaluated which amino acid residues in the C-terminal domain are important for the membrane localization of AQP5. We prepared plasmids expressing hAQP5 point mutants and investigated the subcellular localization of these mutants upon the transfection of HEK-293 cells. Unexpectedly, hAQP5 point mutants with alanine substitutions of Lys^257^, Lys^258^, Thr^259^, Thr^263^, or Thr^264^, phosphorylated or ubiquitinated sites, showed a similar cellular localization pattern compared with hAQP5-WT (Figure 3A). However, and interestingly, hAQP5 point mutants with alanine substitutions of Met^260^, Glu^261^, and Leu^262^ did not. The level of cell surface expression of hAQP5 point mutants, especially L262A, was significantly lower than that of hAQP5-WT (Figure 3B). The overexpression of hAQP5 L262A did not increase the membrane water permeability of CHO-K1 cells (Figure 3C). hAQP5 L262A did not translocalize to the plasma membrane under ionomycin or forskolin treatment, which matched the findings of hAQP5-ΔCT or 1–255 (Figure 3D). Moreover, endocytosis was not involved in the cytosolic localization of hAQP5 L262A (Figure 3E,F). These results suggested that Leu^262^ is important for the plasma membrane localization of AQP5.

### 2.4. C-Terminal Domain Mutant of AQP5 Localized to Autophagosome or Lysosome and Was Degraded by Autophagy

**Figure 4:**
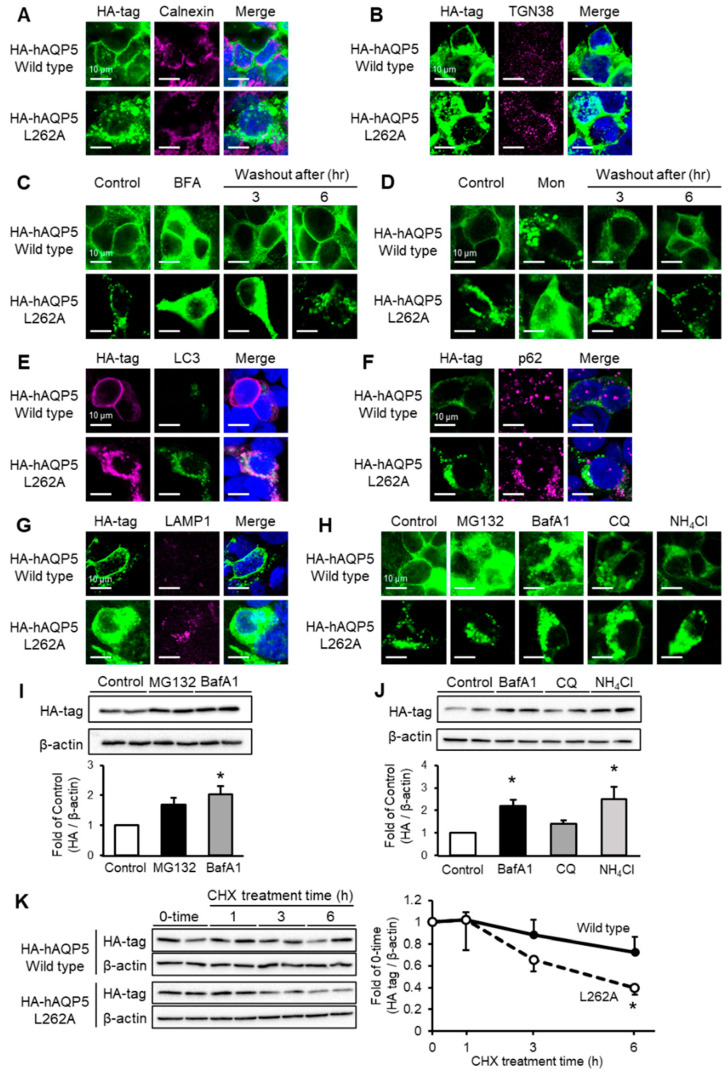
C-terminal domain mutant of AQP5 localizes to autophagosome or lysosome and is degraded via autophagy. HEK-293 cells transfected with hAQP5 wild-type or L262A were analyzed for the cellular localization of the mutants, calnexin (**A**) or TGN38 (**B**), by immunofluorescence. HEK-293 cells transfected with hAQP5 wild-type or L262A were treated with brefeldin A ((**C**), BFA, 5 µg/mL) or monensin ((**D**), Mon, 10 µM) for 12 h, after which the cells were washed out and incubated for 3 or 6 h. The cellular localization of the mutants was assessed by immunofluorescence. HEK-293 cells transfected with hAQP5 wild-type or L262A with LC3-GFP were analyzed for the cellular localization of the mutants and LC3 by immunofluorescence (**E**). HEK-293 cells transfected with hAQP5 wild-type or L262A were analyzed for the cellular localization of the mutants, p62 (**F**), or LAMP1 (**G**), by immunofluorescence. HEK-293 cells transfected with hAQP5 L262A were treated with MG132 (10 µM), bafilomycin A1 (BafA1, 500 nM), chloroquine (CQ, 20 µM), or ammonium chloride (10 µM) for 12 h. The cellular localization of the mutants was analyzed by immunofluorescence (**H**). The level of whole-cell mutants was analyzed by Western blotting. Each data point represents mean ± SE (*n* = 3), * *p* < 0.05 vs. control (**I**,**J**). HEK-293 cells transfected with hAQP5 wild-type and L262A were treated with cycloheximide (100 µg/mL) for 1, 3, 6, or 12 h. The level of whole-cell mutants was analyzed by Western blotting. Each data represents mean ± SE (*n* = 4), * *p* < 0.05 vs. control (**K**). Typical data in triplicated experiments are shown.

Membrane protein synthesis is mediated by peptide synthesis in ribosomes, folding in the endoplasmic reticulum, glycosylation in the Golgi apparatus, and plasma membrane trafficking by secretory vesicles. We hypothesized that the cytosolic localization of hAQP5 L262A is caused by abnormalities in this synthesis pathway. To evaluate whether hAQP5 L262A accumulated in the ER or Golgi, we performed double immunofluorescence for the HA tag and ER/Golgi markers, and stop-and-release assays using brefeldin A (BFA) or monensin (Mon), inhibitors of cis- or trans-Golgi transportation. In hAQP5 L262A-transfected HEK-293 cells, the mutant did not colocalize with calnexin (Figure 4A), the ER marker, or trans-Golgi network 38 (TGN38) (Figure 4B), a Golgi marker. Treatment with BFA or Mon changed the localization of hAQP5 L262A, while washout reversed this change (Figure 4C,D). These results suggested that hAQP5 L262A passed through the ER and Golgi. Autophagy is a process of achieving protein homeostasis by acting on aggregated proteins, so we evaluated whether hAQP5 L262A was degraded by autophagy. Interestingly, hAQP5 L262A colocalized with LC3 (Figure 4E) or p62 (Figure 4F), which are autophagosome markers, in the cytosol. hAQP5 L262A also colocalized with lysosome-associated membrane protein 1 (LAMP1) (Figure 4G), a lysosome marker, and treatment with a lysosome inhibitor (bafilomycin A1, chloroquine, or ammonium chloride) led to the accumulation of hAQP5 L262A in the cytosol, while that of MG132, a proteasome inhibitor, did not (Figure 4H). The level of whole-cell expression of hAQP5 L262A increased with lysosome inhibitor treatment (Figure 4I,J) and the protein stability of hAQP5 L262A decreased markedly compared with that of hAQP5-WT (Figure 4K). These findings together indicate that the C-terminal domain mutants of AQP5 localized in the autophagosome or lysosome and were degraded via autophagy.

## 3. Discussion

The major finding of this study is that the C-terminal domain of AQP5 is required for this protein to avoid degradation at the post-trans-Golgi exocytic pathway, which may be a kind of quality control of this membrane protein. This idea is supported by four lines of evidence. First, AQP5 C-terminal domain mutants did not localize on the plasma membrane (Figure 1A,B, Figure 2A,B and Figure 3A,B). Second, these mutants were not arrested in the ER and Golgi but instead passed through these organelles (Figure 4A–D). Third, endocytosis was shown not to be involved in the abnormal localization of these mutants (Figure 1E,F, Figure 2E,F and Figure 3E,F). Finally, the protein stability of the mutants was significantly decreased compared with that of the WT (Figure 4K). This mechanism of quality control is consistent with that previously reported for the C-terminal domain of AQP2 [22].

The second finding of this study is that AQP5 C-terminal mutants are degraded by autophagy, which is supported by two lines of evidence. First, the AQP5 C-terminal domain mutants colocalized with p62, LC3, or LAMP1, markers of autophagy, in the cytosol (Figure 4E–G). Second, the AQP5 C-terminal domain mutant accumulated upon treatment with bafilomycin A1, chloroquine, or ammonium chloride, which are autophagy inhibitors, but not with MG132, a proteasome inhibitor (Figure 4H–J). Autophagy is integrated into the quality control of some proteins in the ER (ER-phagy) [23,24]. Therefore, the autophagy observed with AQP5 mutants may be an alternative quality control, which is independent of the ER.

Phosphorylation or ubiquitination regulates the localization of many other membrane proteins including other AQP isoforms [25]. For example, phosphorylation of Ser^256^ was reported to facilitate the trafficking of AQP2 to the plasma membrane, and the ubiquitination of Lys^270^ induced AQP2 endocytosis, with these amino acid residues being located in the C-terminal domain of AQP2 [26,27]. Unexpectedly, the homeostasis of AQP5 plasma membrane localization is regulated by neither phosphorylation nor ubiquitination of the C-terminal domain. This is supported by the data showing that the Ala substitution of Lys or Thr of the AQP5 C-terminal domain did not affect plasma membrane localization (Figure 3A,B). Hasegawa et al. reported that Thr^259^ in the C-terminal domain of AQP5 is a site of phosphorylation by protein kinase A (PKA), but phosphorylation of this amino acid residue did not contribute to AQP5 trafficking to the plasma membrane [28], which is consistent with our results.

Owing to the limitations of gene transfection study, we employed HEK-293 cells, a cell line derived from kidneys and lacking expression of the AQP5 gene. hAQP5 C-terminal mutants also exhibited abnormal localization in HSG cells, a cell line from salivary glands with tissue expressing AQP5, and MLE-12 cells, a cell line from lung epithelia with endogenous expression of AQP5 (Figure S1). Our results show that the quality control mechanism functions in AQP5-expressing tissue such as lung epithelia or salivary glands.

Classical protein quality control is performed in the ER, in which misfolded proteins are recognized, poly-ubiquitinated, and then subjected to proteasomal degradation [29]. Protein quality control also occurs in the Golgi apparatus, in which proteins that escape quality control in the ER are recognized, transported to multivescular bodies (MVB), and subjected to lysosomal degradation [30,31,32]. The finding that the AQP5 C-terminal domain mutants were degraded by autophagy may rule out the involvement of protein quality control in the Golgi in explaining our results.

Intracellular uptake mediated by endocytosis is a major pathway for the abnormal cytosolic localization of membrane proteins [33]. For example, cystic fibrosis transmembrane conductance regulator (CFTR) mutant Δ508 is ubiquitinated and internalized by endocytosis [34,35], which causes cystic fibrosis. However, our findings show that endocytosis is not involved in the cytosolic localization of hAQP5 C-terminal domain mutants. This is supported by the findings that hAQP5 C-terminal domain mutants did not colocalize with EEA1, an early endocytosis marker, and did not change their cytosolic localization upon treatment with an endocytosis inhibitor. In good agreement with the results, all hAQP5 deletion mutants were located in the cytosol, indicating that there is no region in the hAQP5 C-terminal domain that promotes intracellular uptake.

In this study, we evaluated the subcellular localization of hAQP5 C-terminal domain mutant in cells cultured in cell culture dishes or on glass slides. However, in AQP5-expressing tissues, receptors and channels localize with apical and basolateral polarity [36]. The N-terminal domain of AQP3 [37] or C-terminal domain of AQP4 [38] was shown to be crucial for their basolateral-polarized localization. We investigated the cellular localization of hAQP5 C-terminal domain mutants in polarized MDCK cells; the mutants did not localize on the plasma membrane as in cells on culture dishes, while wild-type hAQP5 localized in the apical-side membrane (Figure S2). Taking these findings together, the abnormal localization of AQP5 C-terminal domain mutants also takes place in cells with apical–basolateral polarity.

AQPs form a homo-tetramer on the plasma membrane [39]. The C-terminal domain of AQP5 localizes on the outer side of this tetramer, which allows the domain to interact with other proteins in the cytosol. ^262^Leu, a hydrophobic amino acid, is critical for the localization of AQP5 on the plasma membrane. Moreover, cells cotransfected with hAQP5 C-terminal mutant and wild-type AQP5 showed localization of the mutant in the plasma membrane (data not shown). This suggests that interaction between cytoplasmic proteins and at least one monomer of the tetrameric structures causes the trafficking of AQP5 to the plasma membrane. Further studies, such as in silico simulation, are clearly needed to elucidate this idea.

In summary, in this study we demonstrated that the C-terminal domain is required for AQP5 to pass through protein quality control and be trafficked to the plasma membrane. A single amino acid residue is crucial for this quality control mechanism, the mechanism of which may differ from the conventional ones.

## 4. Materials and Methods

### 4.1. Cell Culture

HEK-293T (ATCC CRL-3216) or CHO-K1 (ATCC CCL-61) cells were cultured in Dulbecco’s modified Eagle’s medium (DMEM) (Nissui Pharmaceuticals, Tokyo, Japan) supplemented with 10% fetal bovine serum (FBS) (Hyclone^®^; GE Healthcare Life Sciences, Uppsala, Sweden), 1 unit/mL penicillin, and 1 µg/mL streptomycin (Gibco, Grand Island, NY, USA).

### 4.2. Antibodies

Rabbit anti-HA antibody (600-401-384) was purchased from Rockland Immunochemicals (Gilbertsville, PA, USA). Mouse anti-HA antibody (16B12) was purchased from Biolegend (San Diego, CA, USA). Mouse anti-calnexin (AF18), EEA1 (E-8), TGN38 (B-6), and rat anti-LAMP1 (1D4B) antibodies were purchased from Santa Cruz Biotechnology (Santa Cruz, CA, USA). Rabbit anti-p62 antibody (PM045) was purchased from MBL (Nagoya, Japan). Mouse anti-β-actin antibody (A5441) and horseradish peroxidase (HRP)-conjugated rabbit anti-mouse IgG antibody (A9044) were purchased from Sigma Aldrich (St. Louis, MO, USA). HRP-conjugated donkey anti-rabbit IgG antibody (711-035-152) was purchased from Jackson ImmunoReseach Laboratories (West Grove, PA, USA). Alexa Fluor 488 (A11008)- and 647 (A21244)-conjugated goat anti-rabbit IgG antibody and Alexa Fluor 647-conjugated anti-mouse IgG antibody (A21235) were purchased from Invitrogen (Carlsbad, CA, USA).

### 4.3. Immunofluorescence

Cells cultured on poly-D-lysine-coated glass slides were fixed with 4% paraformaldehyde (Nacalai Tesque, Kyoto, Japan), permeabilized with 0.1% Triton X-100 (MP Biomedicals, Santa Ana, CA, USA) for 30 min, and blocked with 1% bovine serum albumin (Wako, Tokyo, Japan) for 30 min. The cells were then incubated with mouse anti-HA antibody (1:200 dilution), followed by Alexa Fluor 488-conjugated goat anti-rabbit IgG and Alexa Fluor 647-conjugated goat anti-mouse IgG (1:1000 dilution). The cells were mounted in VECTASHIELD mounting medium containing DAPI (Vector Laboratories, Burlingame, CA, USA). All images were taken with a confocal microscope (TCS SP8; Leica Microsystems, Wetzlar, Germany).

### 4.4. Cell Surface Biotinylation Assay

To evaluate AQP5 mutant expression on the cell surface, a cell surface biotinylation experiment was performed as previously reported [11]. Briefly, cell monolayers were washed and incubated with a cell-impermeable biotin derivative (0.5 mg/mL EZ Link Sulfo-NHS-LC-Biotin; Thermo Fisher Scientific, Waltham, MA, USA) in PBS for 30 min to biotinylate cell surface proteins. Unreacted biotinylated molecules were quenched by the addition of 100 mM glycine. Cells were lysed with RIPA buffer (50 mM Tris, pH 7.5, 150 mM NaCl, 1% (*v*/*v*) NP-40, 0.5% (*w*/*v*) deoxycholate, 0.1% (*w*/*v*) SDS, 5 mM EDTA, and 1% (*v*/*v*) proteinase inhibitor cocktail), and immunoprecipitations were performed using streptavidin beads (Thermo Fisher Scientific) to precipitate biotinylated proteins. Beads were washed four times in RIPA buffer as described above and bound proteins were eluted with SDS-PAGE sample buffer (10 mM Tris, 3% (*w*/*v*) SDS, 10% (*v*/*v*) glycerol, 0.01% (*w*/*v*) bromophenol blue, and 2.5% 2-mercaptoethanol) for 30 min at 37 °C. Changes in the amount of biotinylated AQP5 mutants were analyzed by immunoblotting.

### 4.5. Immunoblotting

Cell lysates were separated by 12% SDS-polyacrylamide gel electrophoresis and transferred to PVDF membrane. The membrane was blocked with 5% non-fat dry milk in PBS containing 0.1% Tween 20 (PBS-T) at room temperature for 1 h and incubated with mouse anti-HA antibody (dilution 1:1000) at 4 °C overnight. The membrane was then washed with PBS-T and incubated with HRP-conjugated rabbit anti-mouse IgG antibody (dilution 1:20,000) at room temperature for 1 h. Immunocomplexes were detected by Super Signal West Pico Plus Maximum Sensitivity Substrate (Thermo Fisher Scientific) and images were obtained using the ChemiDoc XRS+ system (Bio-Rad Laboratories, Hercules, CA, USA). The density of bands was quantified using Image Lab software (version 6.0; Bio-Rad Laboratories).

### 4.6. Assay for Plasma Membrane Osmotic Water Permeability

Water permeability across the plasma membrane was measured using the calcein fluorescence quenching method, as described previously [40]. Briefly, CHO-K1 cells transfected with hAQP5 C-terminal domain mutant were cultured on a 96-well, clear-bottomed black plate and loaded with 5 µM Calcein-AM (Dojindo, Tokyo, Japan) for 90 min. Then, cells were washed twice and fluorescence change (λ_ex_ 485 nm, λ_em_ 535 nm) caused by the addition of an equal volume of 300 mM D-(−)-mannitol was measured with a fluorescence plate reader (ARVO X4; PerkinElmer, Norwalk, CT, USA).

### 4.7. Statistical Analysis

All data are expressed as mean ± standard error of the mean (S.E.M.). The significance of differences was assessed using one-way ANOVA followed by the Student–Newman–Keuls test. For all experiments, *p*-values of <0.05 were regarded as significant.

## Figures and Tables

**Table 1:** The amino acid sequence of HA-hAQP5 C-terminal deletion mutants.

Name	221 224 235 245 255 265
HA-hAQP5-WT	-YFYLLFPNSLSLSERVAIIKGTYEPNENWEEQREERKKTMELTTR
HA-hAQP5 1-255	-YFYLLFPNSLSLSERVAIIKGTYEPNENWEEQREE
HA-hAQP5 1-245	-YFYLLFPNSLSLSERVAIIKGTYEP
HA-hAQP5 1-235	-YFYLLFPNSLSLSER
HA-hAQP5 1-224	-YFYL

## Supplementary Materials

The following are available online at https://www.mdpi.com/article/10.3390/ijms222413461/s1.
