# Correction: Muroi, S.-i.; Isohama, Y. C-Terminal Domain of Aquaporin-5 Is Required to Pass Its Protein Quality Control and Ensure Its Trafficking to Plasma Membrane. *Int. J. Mol. Sci.* 2021, *22*, 13461

**DOI:** 10.3390/ijms27010223

**Published:** 2025-12-25

**Authors:** Shin-ichi Muroi, Yoichiro Isohama

**Affiliations:** Laboratory of Applied Pharmacology, Faculty of Pharmaceutical Sciences, Tokyo University of Science, 2641 Yamazaki, Noda 278-8510, Japan; shin36000903@gmail.com

In the original publication [1], there was an error in Figures 2A,B,D–F, 3D–F and 4H as published. The authors sincerely apologize for this oversight and have confirmed that these errors resulted from the inadvertent use of images. The corrected versions of Figure 2, Figure 3 and Figure 4 are provided below. This correction was approved by the Academic Editor. The original publication has also been updated.

## Figures and Tables

**Figure 2:**
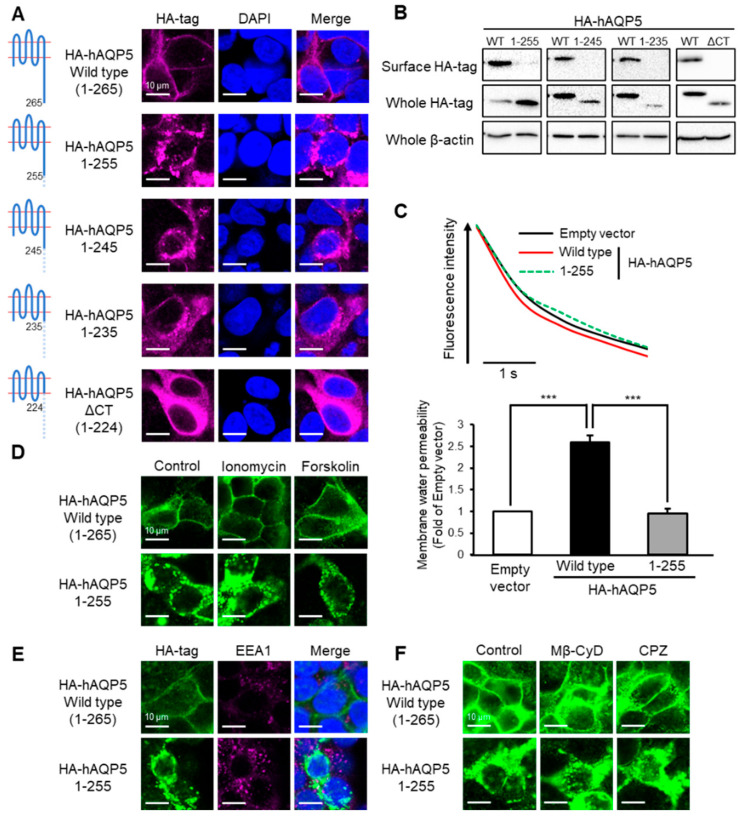
Ten amino acid residues between Arg^256^ and Arg^265^ are required for plasma membrane localization of AQP5. HEK-293 cells transfected with hAQP5 C-terminal deletion mutants were analyzed for the cellular localization of the mutants by immunofluorescence (**A**) and assessed for the level of cell surface mutants by cell surface biotinylation and Western blotting (**B**). Membrane water permeability of CHO-K1 cells transfected with hAQP5 1–255 was measured using the calcein quenching method at 37 °C. Each data point represents mean ± SE (*n* = 3), *** *p* < 0.001 (**C**). HEK-293 cells transfected with hAQP5 1–255 were treated with ionomycin (1 µM) or forskolin (10 µM) for 15 min. The cellular localization of the mutants was analyzed by immunofluorescence (**D**). HEK-293 cells transfected with hAQP5 1–255 were analyzed for the cellular localization of the mutants and EEA1 by immunofluorescence (**E**). HEK-293 cells transfected with hAQP5 1–255 were treated with methyl-β-cyclodextrin (Mβ-CyD, 1 mM) or chlorpromazine (CPZ, 10 µg/mL) for 12 h. The cellular localization of the mutants was analyzed by immunofluorescence (**F**). Typical data in triplicated experiments are shown.

**Figure 3:**
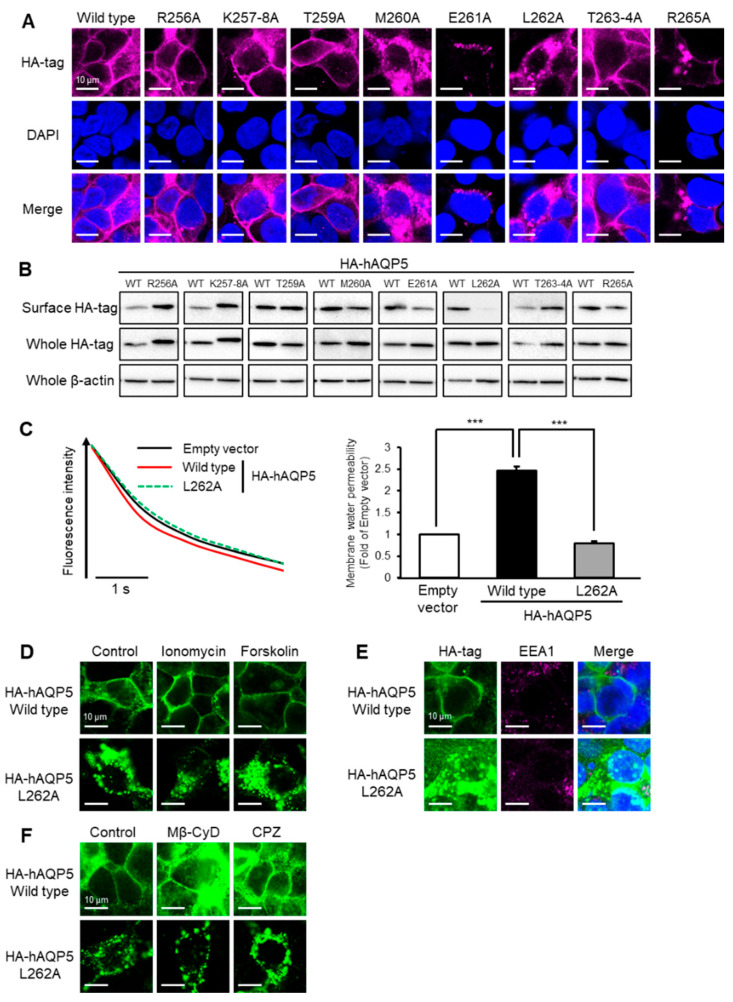
Leu^262^ is required for plasma membrane localization of AQP5. HEK-293 cells transfected with hAQP5 C-terminal point mutants were analyzed for the cellular localization of the mutants by immunofluorescence (**A**) and assessed for the level of cell surface mutants by cell surface biotinylation and Western blotting (**B**). Membrane water permeability of CHO-K1 cells transfected with hAQP5 L262A was measured using the calcein quenching method at 37 °C. Each data point represents mean ± SE (*n* = 3), *** *p* < 0.001 (**C**). HEK-293 cells transfected with hAQP5 L262A were treated with ionomycin (1 µM) or forskolin (10 µM) for 15 min. The cellular localization of the mutants was analyzed by immunofluorescence (**D**). HEK-293 cells transfected with hAQP5 L262A were analyzed for the cellular localization of the mutants and EEA1 by immunofluorescence (**E**). HEK-293 cells transfected with hAQP5 L262A were treated with methyl-β-cyclodextrin (Mβ-CyD, 1 mM) or chlorpromazine (CPZ, 10 µg/mL) for 12 h. The cellular localization of the mutants was analyzed by immunofluorescence (**F**). Typical data in triplicated experiments are shown.

**Figure 4:**
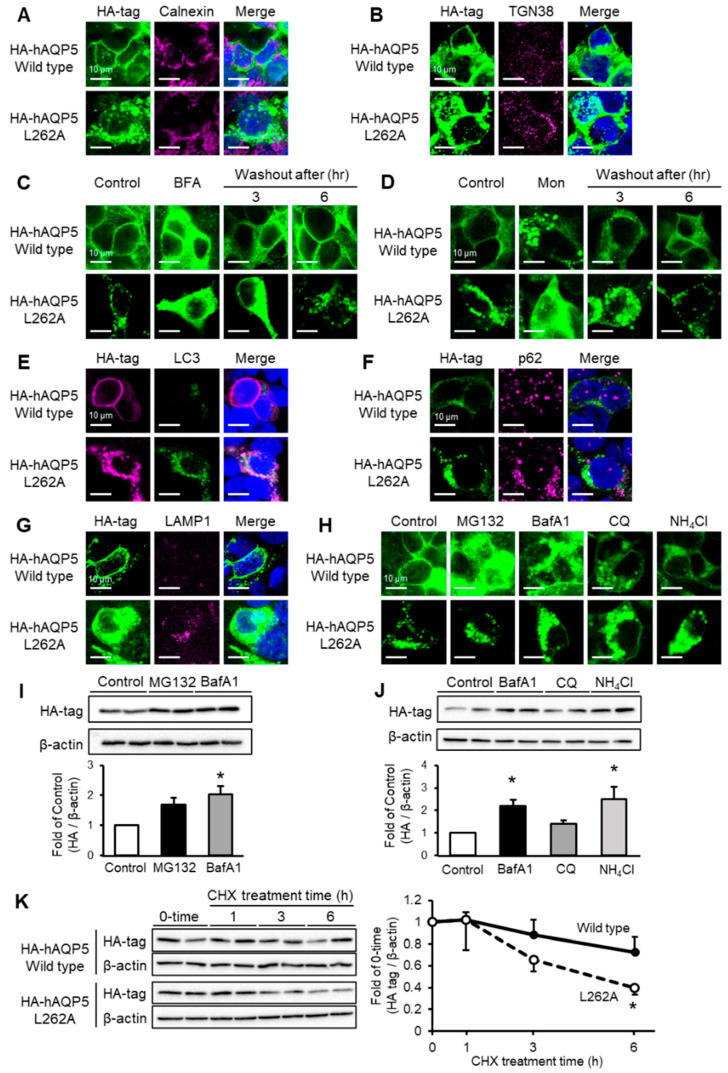
C-terminal domain mutant of AQP5 localizes to autophagosome or lysosome and is degraded via autophagy. HEK-293 cells transfected with hAQP5 wild-type or L262A were analyzed for the cellular localization of the mutants, calnexin (**A**) or TGN38 (**B**), by immunofluorescence. HEK-293 cells transfected with hAQP5 wild-type or L262A were treated with brefeldin A ((**C**), BFA, 5 µg/mL) or monensin ((**D**), Mon, 10 µM) for 12 h, after which the cells were washed out and incubated for 3 or 6 h. The cellular localization of the mutants was assessed by immunofluorescence. HEK-293 cells transfected with hAQP5 wild-type or L262A with LC3-GFP were analyzed for the cellular localization of the mutants and LC3 by immunofluorescence (**E**). HEK-293 cells transfected with hAQP5 wild-type or L262A were analyzed for the cellular localization of the mutants, p62 (**F**), or LAMP1 (**G**), by immunofluorescence. HEK-293 cells transfected with hAQP5 L262A were treated with MG132 (10 µM), bafilomycin A1 (BafA1, 500 nM), chloroquine (CQ, 20 µM), or ammonium chloride (10 µM) for 12 h. The cellular localization of the mutants was analyzed by immunofluorescence (**H**). The level of whole-cell mutants was analyzed by Western blotting. Each data point represents mean ± SE (*n* = 3), * *p* < 0.05 vs. control (**I**,**J**). HEK-293 cells transfected with hAQP5 wild-type and L262A were treated with cycloheximide (100 µg/mL) for 1, 3, 6, or 12 h. The level of whole-cell mutants was analyzed by Western blotting. Each data represents mean ± SE (*n* = 4), * *p* < 0.05 vs. control (**K**). Typical data in triplicated experiments are shown.
