# Supplementary material for: C-Terminal Domain of Aquaporin-5 Is Required to Pass Its Protein Quality Control and Ensure Its Trafficking to Plasma Membrane
Source: Int J Mol Sci. 2021 Dec 15;22(24):13461. doi: 10.3390/ijms222413461 (PMC8707437; doi:10.3390/ijms222413461)
Supplement: Supplementary file 1 [file ijms-22-13461-s001.zip › ijms-1478304-supplementary.pdf]

International Journal of
Molecular Sciences

MDPI

Article

C-Terminal Domain of Aquaporin-5 Is Required to Avoid Its Protein Quality Control and Ensure Its Trafficking to Plasma Membrane

Shin-ichi Muroi and Yoichiro Isohama *

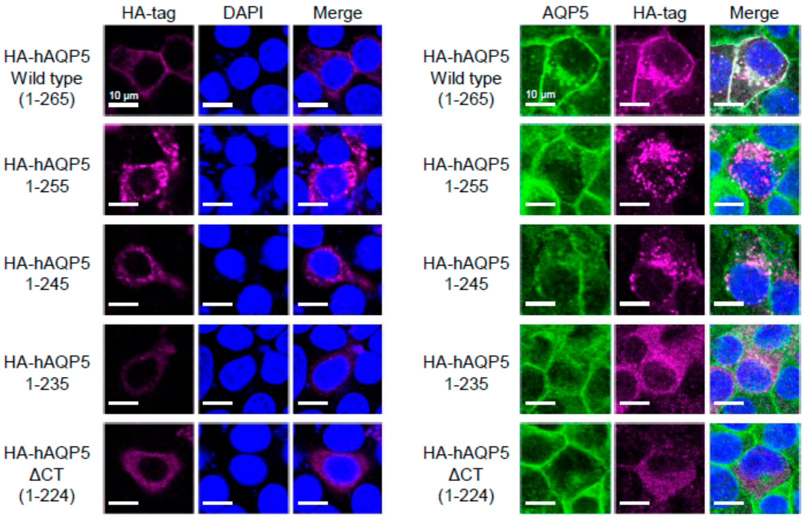

Figure S1. C-terminal domain is required for plasma membrane localization of AQP5 in HSG or MLE-12 cells. HSG (A) or MLE-12 (B) cells transfected with hAQP5 C-terminal deletion mutants were analyzed.

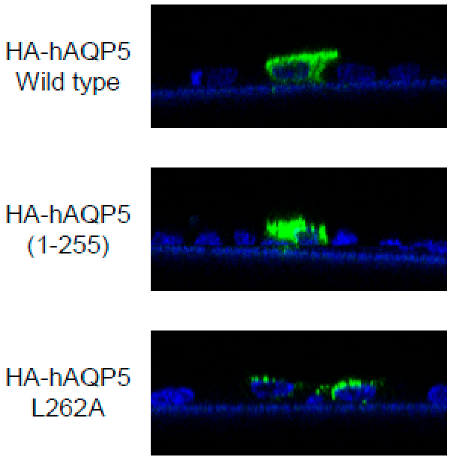

Figure S2. C-terminal domain is required for plasma membrane localization of AQP5 in polarized MDCK cells. MDCK cells were transfected with hAQP5 C-terminal deletion mutants and cultured until confluent on a Transwell. The cellular localization of the mutants was analyzed by immunofluorescence.
